# The Ubiquitin E3 Ligase PUB17 Positively Regulates Immunity by Targeting a Negative Regulator, KH17, for Degradation

**DOI:** 10.1016/j.xplc.2020.100020

**Published:** 2020-01-07

**Authors:** Hazel McLellan, Kai Chen, Qin He, Xintong Wu, Petra C. Boevink, Zhendong Tian, Paul R.J. Birch

**Affiliations:** 1 Division of Plant Science, School of Life Science, University of Dundee (at JHI), Invergowrie, Dundee DD2 5DA, UK; 2 Key Laboratory of Horticultural Plant Biology (HZAU), Ministry of Education, Key Laboratory of Potato Biology and Biotechnology (HZAU), Ministry of Agriculture and Rural Affairs, Huazhong Agricultural University, Wuhan, Hubei 430070, China; 3 Cell and Molecular Science, James Hutton Institute, Invergowrie, Dundee DD2 5DA, UK

**Keywords:** oomycete, plant disease, late blight, E3 ligase, KH RNA-binding protein

## Abstract

Ubiquitination is a post-translational modification that regulates many processes in plants. Several ubiquitin E3 ligases act as either positive or negative regulators of immunity by promoting the degradation of different substrates. StPUB17 is an E3 ligase that has previously been shown to positively regulate immunity to bacteria, fungi and oomycetes, including the late blight pathogen *Phytophthora infestans*. Silencing of *StPUB17* promotes pathogen colonization and attenuates Cf4/avr4 cell death. Using yeast-2-hybrid and co-immunoprecipitation we identified the putative K-homology (KH) RNA-binding protein (RBP), StKH17, as a candidate substrate for degradation by StPUB17. StKH17 acts as a negative regulator of immunity that promotes *P. infestans* infection and suppresses specific immune pathways. A KH RBP domain mutant of StKH17 (StKH17^GDDG^) is no longer able to negatively regulate immunity, indicating that RNA binding is likely required for StKH17 function. As StPUB17 is a known target of the ubiquitin E3 ligase, StPOB1, we reveal an additional step in an E3 ligase regulatory cascade that controls plant defense.

## Teaser

Ubiquitination is a post-translational modification that controls many aspects of plant growth, development, and stress responses. The ubiquitin E3 ligase PUB17 is a positive regulator of immunity. This study reveals that PUB17 interacts with a K-homology RNA-binding protein, KH17, which negatively regulates immunity. Interaction of PUB17 with KH17 results in their co-degradation by the 26S proteasome, promoting the activation of specific immune responses.

## Introduction

Plants are constantly subjected to attack by microbes in the environment. However, they have evolved a sensitive two-tier surveillance system that is able to recognize and thwart most attempted incursions. The first layer of defense comprises recognition of conserved microbe-associated molecular patterns (MAMPs) by cell surface pattern recognition receptors (PRRs). This up-regulation of immune responses is termed pattern-triggered immunity (PTI) and prevents infection by most microbes (Jones and Dangl, 2006). Host-adapted pathogens are able to suppress PTI through the activity of secreted effector proteins that can manipulate immunity; this is called effector-triggered susceptibility (ETS). The second layer of plant defenses involves the detection of these effectors, or their activities, by plant resistance (R) genes. This recognition results in a massively amplified defense response termed effector-triggered immunity (ETI), which can halt pathogen colonization (Jones and Dangl, 2006).

The plant immune responses can include the synthesis of antimicrobial compounds and defense hormones, cell wall reinforcement, generation of reactive oxygen intermediates (ROIs), and a form of programmed cell death (PCD) called the hypersensitive response (HR) (Dixon et al., 1994). While regulation of immunity requires huge alterations to the transcriptome (Li et al., 2016), changes in post-translational modifications (PTMs) are emerging as an important means of controlling and coordinating defense responses. One such PTM is ubiquitination, which involves the covalent attachment of ubiquitin (Ub) to a lysine residue in the protein of interest. There are three enzymes needed for ubiquitination. An E1 activating enzyme is required to recruit Ub; an E2 conjugating enzyme, which determines Ub transfer and type of Ub linkage; and an E3 ligase, which is responsible for selecting substrates for ubiquitination (Sadanandom et al., 2012). Ubiquitination is a reversible process and there is a family of deubiquitinating enzymes (DUBs) that remove Ub (Isono and Nagel, 2014). The precise form of ubiquitination (i.e., monoubiquitination or polyubiquitination) and the type of linkages in the Ub chain formation can specify different fates for the substrate; for example, by causing changes in localization or activity (Chen and Sun, 2009). However, the major mode of action is the addition of a polyubiquitin chain to target the substrate for degradation by the 26S proteasome. Ubiquitination has been shown to regulate many different processes in plants, from growth and development, including flowering, through to responses to both abiotic and biotic stresses (Sharma et al., 2016). One interesting observation is that there has been a considerable proliferation in the number and type of E3 ligases in plants compared to animals (Vierstra, 2003), indicating the relative importance of ubiquitination as a regulatory mechanism. There are several families of E3 ligases in plants with their classification based on their protein domains: these are homology to E6-Ap C terminus (HECT) domains, plant ubox (PUB) domains, and really interesting new gene (RING) domains, with the latter group divided into those that work as monomers and those that work as part of a cullin-based E3 ligase complex (Chen and Hellmann, 2013).

Many E3 ligases act as negative regulators of plant defense. For example, PUB12 and PUB13 work together to ubiquitinate the flg22 receptor FLS2, resulting in its degradation (Lu et al., 2011). PUB13 is also able to down-regulate SA-dependent pathogenesis-related gene expression through interactions with RabA4B and phosphatidylinositol 4-kinase β (PI4Kβ1/2) (Antignani et al., 2015). SPL11 is a PUB E3 ligase from rice that has some similarity to PUB13. Spl11 mutants behave as lesion mimics with runaway cell death and heightened defense gene activation suggesting SPL11 negatively regulates immunity (Zeng et al., 2004). SPL11 has been reported to ubiquitinate the RhoGAP protein SPL11-interacting Protein 6 (SPIN6) for degradation by the proteasome (Liu et al., 2015). *Arabidopsis* PUB22/23/24 act redundantly to suppress PTI signaling, including ROI production and mitogen-activated protein kinase kinase kinase (MAP3K) activation (Trujillo et al., 2008), with PUB22 shown to ubiquitinate exocyst subunit Exo70B2, a positive regulator of PTI, targeting it for degradation by the 26S proteasome (Stegmann et al., 2012). The cullin-based E3 ligase BTB domain containing NPH3/RPT2-LIKE1 protein (NRL1) is a negative regulator of immunity and a susceptibility (S) factor (Yang et al., 2016). NRL1 is manipulated by *Phytophthora infestans* effector Pi02860, by promoting the proteasome-mediated degradation of a positive regulator, guanine nucleotide exchange factor SWAP70 (He et al., 2018). Two additional BTB-domain-containing proteins, nonexpresser of PR genes (NPR) 3 and NPR4, are also part of a cullin-based E3 ligase complex. They negatively regulate SA-associated immunity by targeting paralogue NPR1, a positive regulator, for degradation by the 26S proteasome (Fu et al., 2012). The POZ/BTB-containing-protein 1 (POB1) acts with paralogue POB2 to negatively regulate defense to *Botrytis cinerea* and *Hyaloperonospora arabidopsidis* (Qu et al., 2010). Moreover, POB1 has been shown to negatively regulate various HRs and defense to *P. infestans* by promoting the proteasome-mediated degradation of the positive regulator of defense, E3 ligase PUB17 (Orosa et al., 2017).

While E3 ligases such as PUB17 act as positive regulators of defense, the substrates targeted by these positive regulators are as yet unknown. PUB17 is required for selected HRs and resistance to a range of pathogens from different kingdoms, including bacteria, fungi, and the oomycete *P. infestans* (Yang et al., 2006, He et al., 2015). PUB20/CMPG1 is required for an overlapping but distinct set of PCD-promoting pathways, as well as being the target of *P. infestans* effector Avr3a (Gonzalez-Lamothe et al., 2006, Bos et al., 2010, Gilroy et al., 2011). StRFP1 and NbATL60 are MAMP-responsive RING-type E3 ligases, which positively regulate PTI and defense to *P. infestans* (Zhong et al., 2018). Finally, the potato ubox and kinase (StUBK) E3 ligase, a target of *P. infestans* effector PiSFI3, positively regulates immunity to *P. infestans* and flg22 signaling but has no known involvement in PCD (He et al., 2019).

Another area that is emerging as central to control of plant immunity involves RNA-binding proteins (RBPs). These typically form riboprotein complexes with RNA and thereby regulate the translation, stability, and transport of defense-associated RNAs, as well as aspects of gene silencing (Staiger et al., 2013, Hentze et al., 2018). Glycine-rich RNA-binding protein 7 (AtGRP7) is an RNA recognition motif (RRM)-type RBP that regulates the stability of its own transcript as well as those of PRRs FLS2 and EFR. AtGRP7 is targeted by the *Pseudomonas syringae* (*Pst*) effector HopU1, which ADP ribosylates the RRM, preventing it binding RNA, resulting in increased *Pst* colonization (Fu et al., 2007; Nicaise et al., 2013). Modifier of snc1 (MOS2) is an RBP that is responsible for the correct splicing of the transcript of suppressor of npr1-1, constitutive1 (SNC1), a TIR NB-LRR gene (Copeland et al., 2013). PSR1-Interacting Protein 1 (PINP1) is an RBP with an RNA helicase domain. It acts to promote immunity by allowing the accumulation of small RNAs and microRNAs. PINP1 is targeted by the *Phytophthora sojae* effector *Phytophthora* Suppressor of RNA Silencing 1 (PSR1), which disrupts the formation of dicer-containing RNA processing complexes in the nucleus (Qiao et al., 2015). The K homology (KH) RBP AtESR1 regulates JA signaling and resistance to fungal pathogen *Fusarium oxysporum* by an unknown mechanism (Thatcher et al., 2015). Finally, the effector Pi04089 from *P. infestans* interacts with and stabilizes the KH RBP StKRBP1, which promotes pathogen colonization of the host (Wang et al., 2015).

The oomycete *P. infestans* is an economically important pathogen of potato, which is the fourth main staple food crop in the world after maize, rice, and wheat (Fry et al., 2015, Yildiz, 2018). Therefore, it is imperative to understand how the plant immune system responds to and is manipulated by *P. infestans* in order to find novel strategies to fight this pathogen. Previous studies have shown that StPUB17 positively regulates defense to a variety of pathogens, including *P. infestans* (Yang et al., 2006, He et al., 2015). As StPUB17 is itself degraded by the CUL3-based E3 ligase and negative regulator of immunity POB1 (Orosa et al., 2017), this places StPUB17 substrates in an E3 ligase cascade that controls defense to *P. infestans*. To this end, yeast-2-hybrid (Y2H) was used to identify the KH RBP StKH17 as a candidate substrate of StPUB17. StKH17 is indeed turned over in the presence of StPUB17 in a proteasome-dependent manner. StKH17 acts as a negative regulator of immunity to *P. infestans* and an intact RNA-binding domain (BD) is required for this activity.

## Results

### The E3 Ligase StPUB17 Interacts with A Putative RNA Binding Protein StKH17

**Figure 1:**
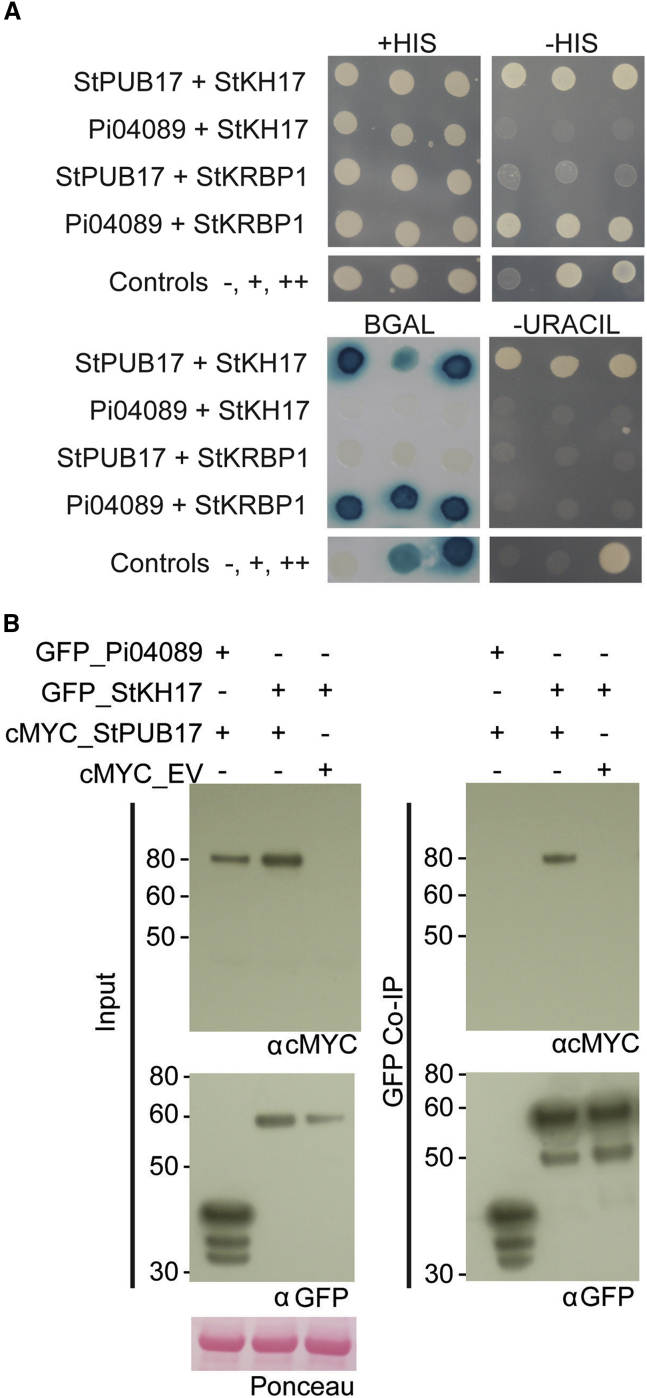
StPUB17 Interacts with StKH17 *In Vitro* and *In Planta*. **(A)** Yeast containing StPUB17 and StKH17 grew on medium lacking histidine (–HIS) or uracil and showed β-galactosidase (BGAL) activity indicating protein-protein interaction. Yeast co-expressing controls Pi04089 and StKRBP1 grow on –HIS and show BGAL activity but there was no activation of any reporters when either was co-expressed with StKH17 or StPUB17. All yeast grew on medium containing histidine (+HIS). The yeast controls are as follows: –, no interaction; +, weak interaction; ++,strong interaction. **(B)** Co-immunoprecipitation assays confirmed the interaction *in planta*. Following pull-downs with GFP-trap beads, GFP-StKH17 associated with cMYC-PUB17 but GFP-Pi04089 did not. Expression of constructs in *N. benthamiana* leaves is indicated by a plus sign (+). Protein size markers are indicated in kilodaltons, and protein loading is indicated by Ponceau stain.

In order to identify putative substrates of the E3 ligase StPUB17, a Y2H screen was conducted against a potato library generated from leaf material infected by *P. infestans* (Bos et al., 2010). Although the screen was carried out to a depth of 2.94 × 10^6^ transformants, only six positive clones were recovered. Five of these encoded a putative RBP with a KH domain and a signal transducer and activator of RNA (STAR) domain (Supplemental Figure 1) corresponding to potato transcript PGSC0003DMT400071249 (hereafter referred to as *StKH17*). In order to confirm this interaction, a GAL4 DNA-BD fusion of StPUB17 was co-transformed into yeast with a GAL4 activation domain (AD) fusion of StKH17. Yeasts containing these constructs were positive in the three reporter gene assays, including the more stringent uracil assay, suggesting that the interaction between the two proteins was strong (Figure 1A). Additional controls, BD-Pi04089 and AD-StKRBP1, respectively comprising a *P. infestans* effector and a KH-type RBP previously shown to interact with each other in yeast and *in planta* (Wang et al., 2015), were also co-transformed into yeast yielding the expected reporter gene activation (Figure 1A). However, co-expression in yeast of BD-Pi04089 with AD-StKH17 failed to activate reporters, as did BD-StPUB17 with AD-StKRBP1, showing that the interaction between PUB17 and KH17 is specific. All yeast grew on the control media containing histidine (Figure 1A). To confirm whether the interaction also occurs *in planta*, co-immunoprecipitation was performed using *Agrobacterium*-mediated transient expression of protein fusions in *Nicotiana benthamiana*, a widely used model host for late blight disease (Whisson et al., 2016). Following incubation of samples with GFP-trap beads, GFP-StKH17 was observed to specifically co-immunoprecipitate cMYC-StPUB17, whereas a GFP-Pi04089 control did not (Figure 1B).

### StPUB17 and StKH17 Interact in the Nucleoplasm

**Figure 2:**
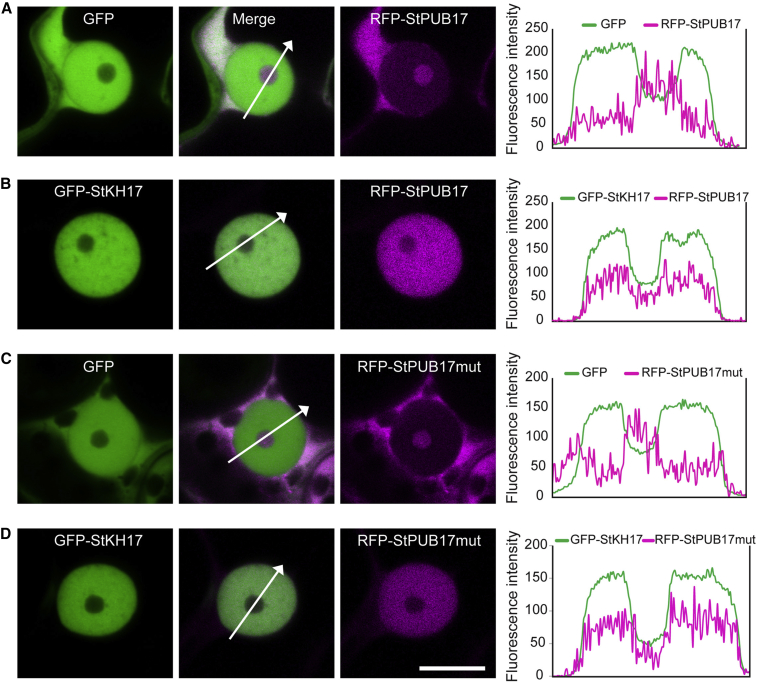
StKH17 and StPub17 Co-localize in the Nucleus and StPUB17 WT and Mutant Forms Are Re-localized from the Nucleolus. **(A–D) (A)** Free GFP with RFP-StPUB17, **(B)** GFP-StKH17 with RFP-StPUB17, **(C)** Free GFP with RFP-StPUB17mut, and **(D)** GFP-STKH17 with RFP-StPUB17mut. Single optical sections through nuclei showing that RFP-StPUB17 WT and mutant are depleted from the nucleolus following co-expression with GFP-StKH17 but not with free GFP. GFP (green) and RFP (magenta) channels are shown separately alongside a merged image. Scale bar represents 10 μm. White arrows indicate the transects for the fluorescence intensity plots shown to the right of each set of images.

StPUB17 has been shown to localize to and act in the nucleus (He et al., 2015). Therefore, the localization of StKH17 was examined using confocal microscopy. GFP-StKH17 was found to accumulate strongly in the nucleoplasm but not in the nucleolus, and showed little or no cytoplasmic background (Supplemental Figure 2), whereas the RFP-StPUB17 wild-type (WT) and ubox-mutant fusion proteins exhibit the same localization as the previously published GFP fusions (He et al., 2015), namely nucleus and nucleolus with cytoplasmic background (Supplemental Figure 2). The dominant-negative ubox domain mutant StPUB17 Val314Ile, Val316Ile, was designed to abolish E3 ligase activity (Yang et al., 2006, He et al., 2015) and is hereafter referred to as StPUB17mut. Co-localization studies were performed using GFP-StKH17 with RFP-StPUB17 or RFP-StPUB17mut constructs. Curiously, upon co-localization with GFP-StKH17 both RFP-StPUB17 and RFP-StPUB17mut constructs no longer accumulate in the nucleolus, although they remain co-localized with GFP-StKH17 in the nucleoplasm (Figure 2). Fluorescence intensity plots drawn through the nucleus show a clear reduction in signal in the area corresponding to the nucleolus in the red channel when GFP-StKH17 is co-expressed with both RFP-StPUB17 and RFP-StPUB17mut. However, both RFP-StPUB17 and RFP-StPUB17mut show a peak in fluorescence intensity corresponding to the nucleolus when co-expressed with a free GFP control, showing that removal from the nucleolus is dependent on the presence of GFP-StKH17 (Figure 2). Analysis carried out using bimolecular fluorescence complementation (BiFC) confirms that YN-StKH17 and YC-StPUB17 interact in the nucleus to reconstitute YFP fluorescence, but are only observed in the presence of the 26S proteasome inhibitor MG132 (Supplemental Figure 3), suggesting that the complex may be turned over by the proteasome.

### The StKH17-StPUB17 Complex Is Degraded in a Proteasome-Dependent Manner

**Figure 3:**
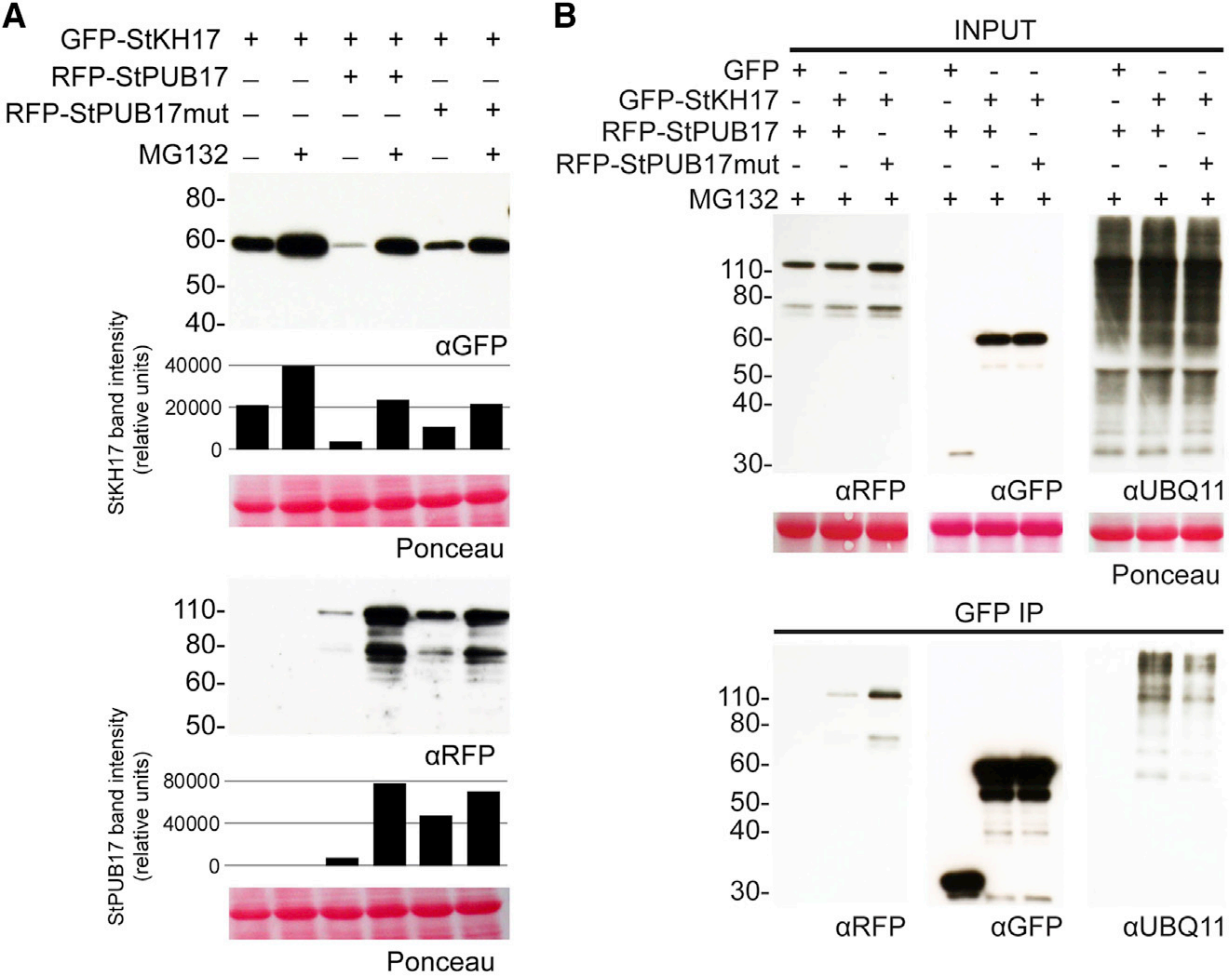
StKH17 is Turned Over in the Presence of StPUB17 in an MG132-Dependent Manner. **(A)** Immunoblots showing that the stability of both GFP-StKH17 and RFP-StPUB17 WT (and to a lesser extent RFP-StPUB17mut) is reduced upon their co-expression and that protein stability is recovered following MG132 treatment. Graphs show band intensity measurements corresponding to the immunoblot panel directly above. **(B)** Ubiquitination assay immunoblots showing strong ubiquitin laddering of GFP-StKH17 in the presence of RFP-StPUB17 following immunoprecipitation (IP) with GFP-trap beads; this ubiquitination is much weaker when GFP-StKH17 is co-expressed with RFP-StPUB17mut. Free GFP is not ubiquitinated by RFP-StPUB17. Expression of constructs or treatment for 6 h with 100 μM MG132 is indicated by a plus sign (+). Protein size markers are indicated in kilodaltons, and protein loading is indicated by Ponceau stain.

As StPUB17 is an ubiquitin E3 ligase, protein stability was assessed to determine if StKH17 is a substrate targeted for degradation by the 26S proteasome. *Agrobacterium* transient expression was used to express GFP-StKH17 and RFP-StPUB17, either alone or together. When expressed together, the stability of StKH17 was reduced and this was at least partially prevented by addition of the proteasome inhibitor MG132 (Supplemental Figure 4). This indicates turnover by the proteasome, which suggests that StKH17 is a substrate of StPUB17. Interestingly, RFP-StPUB17 protein levels mirror those of GFP-StKH17; less stable upon co-expression with GFP-StKH17 while stability is restored by MG132 treatment (Supplemental Figure 4). Thus, it is plausible that the entire StKH17-StPUB17 complex is turned over by the 26S proteasome, as has been observed for several E3 ligase and substrate combinations (reviewed in De Bie and Ciechanover, 2011). Compared with the turnover of GFP-StKH17 when co-expressed with RFP-StPUB17, the turnover rate was considerably reduced when GFP-KH17 was co-expressed with RFP-StPUB17mut, although, it still occurred to some degree (Figure 3A, Supplemental Figure 5A). Again, stability was restored by MG132 treatment.

To provide additional evidence that StKH17 is ubiquitinated in the presence of StPUB17, a ubiquitination assay was carried out. RFP-StPUB17 was co-expressed with either free GFP or GFP-StKH17. RFP-StPUB17mut was also co-expressed with GFP-StKH17. In the input samples, a smear of ubiquitin is detected for all samples, using an ubiquitin antibody. Following a GFP co-immunoprecipitation, distinct ubiquitin laddering of GFP-StKH17 was detected in the presence of StPUB17 but no laddering of the GFP control was observed (Figure 3B, Supplemental Figure 5B). However, in the sample where GFP-StKH17 was co-expressed with RFP-StPUB17mut, some faint ubiquitin laddering of GFP-StKH17 was still detected, consistent with there being some turnover of KH17 when co-expressed with StPUB17mut in the absence of MG132 (Figure 3A). The GFP co-immunoprecipitation (Figure 3B, Supplemental Figure 5B) and Y2H analysis (Supplemental Figure 6) confirm that StPUB17mut is still able to interact directly and strongly with StKH17.

### StKH17 Negatively Regulates Plant Immunity to *P. infestans*

As StPUB17 is a positive regulator of plant immunity to *P. infestans* (Ni et al., 2010, He et al., 2015), and StKH17 behaves as a substrate of StPUB17, the potential involvement of StKH17 in regulating defense to *P. infestans* was investigated. Virus-induced gene silencing (VIGS) was used to transiently silence *NbKH17* in *N. benthamiana* and stable RNAi transgenic lines were produced to silence *StKH17* in potato. Gene expression analysis using quantitative (q)RT–PCR showed a 70%–80% reduction in *NbKH17* levels in *N. benthamiana* plants expressing the independent VIGS constructs TRV-KH17 V1 and TRV-KH17 V2 (Supplemental Figure 7) and an 80%–90% reduction in *StKH17* transcript levels in potato RNAi lines #20, #33, and #34 (Supplemental Figure 8A), compared with the controls TRV-GFP and E potato-3, respectively. No obvious growth or morphological phenotypes were observed in any *KH17*-silenced plants, either transiently in *N. benthamiana* or in stably silenced potato lines (Supplemental Figures 7 and 8), suggesting that the gene does not contribute to development. We also found additional KH-type RBP-encoding genes, *StKH17-like* and *NbKH17-like*, which exist in a distinct cluster to *StKH17* and *NbKH17* based on phylogenetic analysis (Supplemental Figure 8C). However, off-target silencing should not occur as no identical 21 nt stretches exist between *NbKH17-like* and *NbKH17* VIGS constructs, or *StKH17-like* and the *StKH17* RNAi construct (Supplemental Figure 8D–8F).

**Figure 4:**
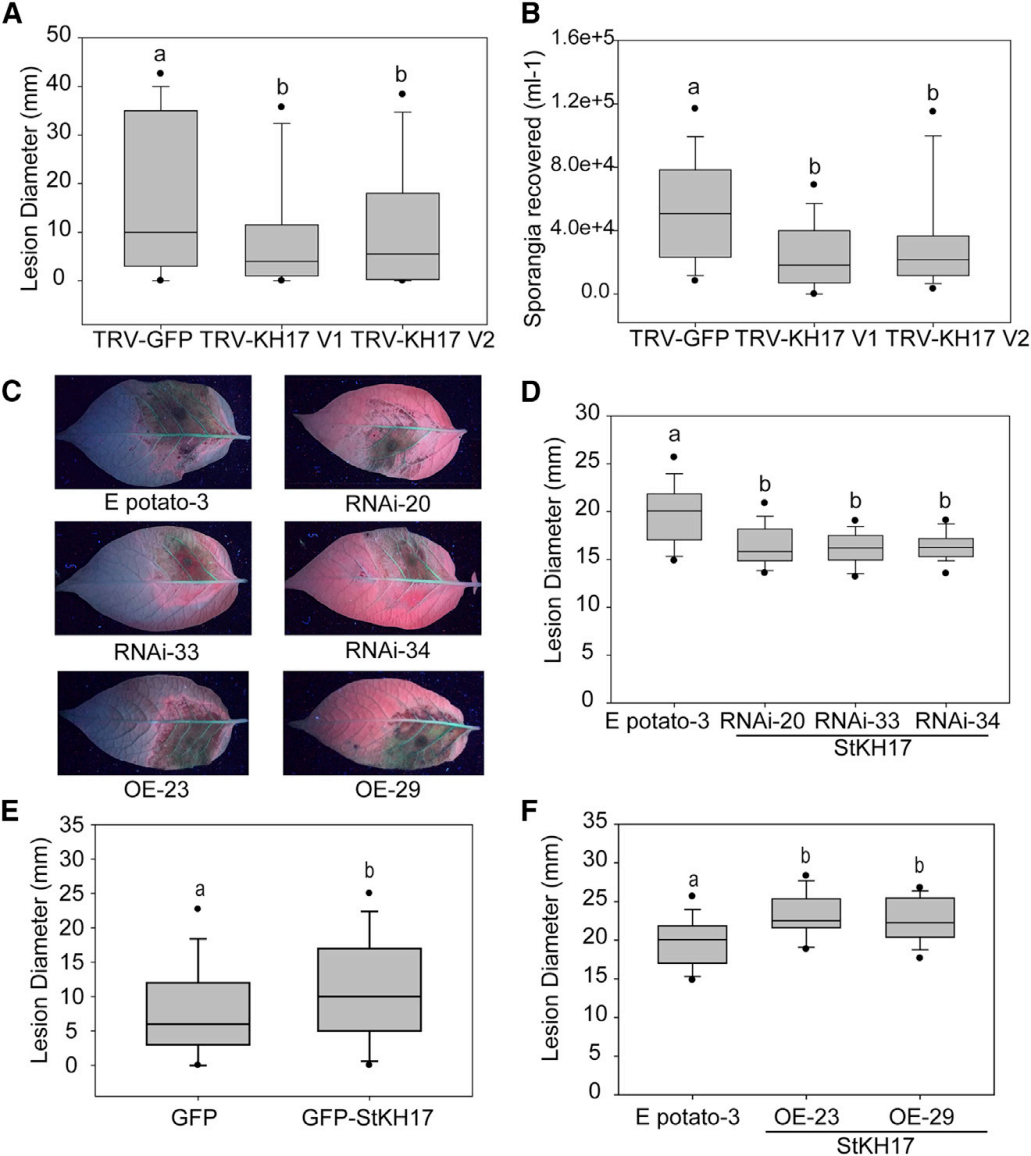
KH17 Silencing by VIGS or Stable RNAi Reduces *P. infestans* Infection while StKH17 Transient or Stable Overexpression Enhances *P. infestans* Colonization. **(A)** Box plot showing lesion diameter is reduced in TRV-KH17-silenced *N. benthamiana* plants compared to the TRV-GFP control (one-way ANOVA p < 0.001, N = 145), The combined data are shown for six biological replicates comprising ~three leaves from ~four plants per replicate. **(B)** Box plot showing the sporangia recovered per milliliter is reduced in TRV-KH17-silenced *N. benthamiana* plants compared to the TRV-GFP control (ANOVA p < 0.001, N = 216). The combined data are shown for six biological replicates comprising ~three leaves from ~four plants per replicate. **(C)** Representative leaf images taken under UV light showing *P. infestans* lesions on E potato-3 control and transgenic potato RNAi and overexpression (OE) lines. **(D)** Box plot showing lesion diameter is reduced in transgenic potato plants silencing StKH17 compared to the E potato-3 control (ANOVA p < 0.001, N = 30). The combined data are shown for three biological replicates comprising ~two leaves from ~five plants per replicate. **(E)** Box plot showing the lesion diameter is increased in the halves of *N. benthamiana* leaves transiently overexpressing GFP-StKH17 compared to those overexpressing free GFP (ANOVA p = 0.006, N = 105). The combined data are shown for three biological replicates comprising ~three leaves from ~six plants per replicate. **(F)** Box plot showing the lesion diameter is increased in transgenic potato plants overexpressing StKH17 compared to the E potato-3 control (ANOVA p < 0.001, N = 30). The combined data are shown for three biological replicates comprising ~two leaves from ~five plants per replicate. Error bars are SD and the median is marked with a horizontal line. Black dots show the 5th and 95th percentile data points. Lowercase letters indicate significant differences tested by one-way ANOVA with pairwise comparisons performed using the Holm-Sidak test.

After challenge with *P. infestans*, a significant reduction in pathogen colonization and lower levels of sporulation were observed on *KH17* VIGS plants (Figure 4A and 4B; Supplemental Figure 7D). In agreement, potato RNAi lines also showed smaller disease lesion sizes compared to the control (Figure 4C and 4D). Reduced pathogen colonization when *KH17* is silenced suggests that StKH17 acts as a negative regulator of immunity to *P. infestans*. To further explore this, overexpression of StKH17 was carried out both in *N. benthamiana* and potato. Transient agroexpression of GFP-StKH17 and a free GFP control in either half of *N. benthamiana* leaves followed by *P. infestans* inoculation resulted in a significant increase in pathogen colonization as observed by increased lesion size in the presence of GFP-StKH17 (Figure 4E). Stable potato transformants overexpressing *StKH17* were also produced, although only two lines showed substantially increased expression (Supplemental Figure 8B). No obvious growth or morphological phenotypes were observed in the potato *StKH17* overexpression lines. Following *P. infestans* infection, significantly increased colonization was also observed on the overexpression lines #23 and #29 compared to the E potato-3 control (Figure 4F). This supports the role of StKH17 as a negative regulator of immunity to *P. infestans* and suggests that the positive regulator of defense, StPUB17, acts by targeting a negative regulator, StKH17, for degradation by the 26S proteasome.

### Overexpression of *StKH17* Specifically Suppresses Cf4/Avr4-Induced Cell Death

**Figure 5:**
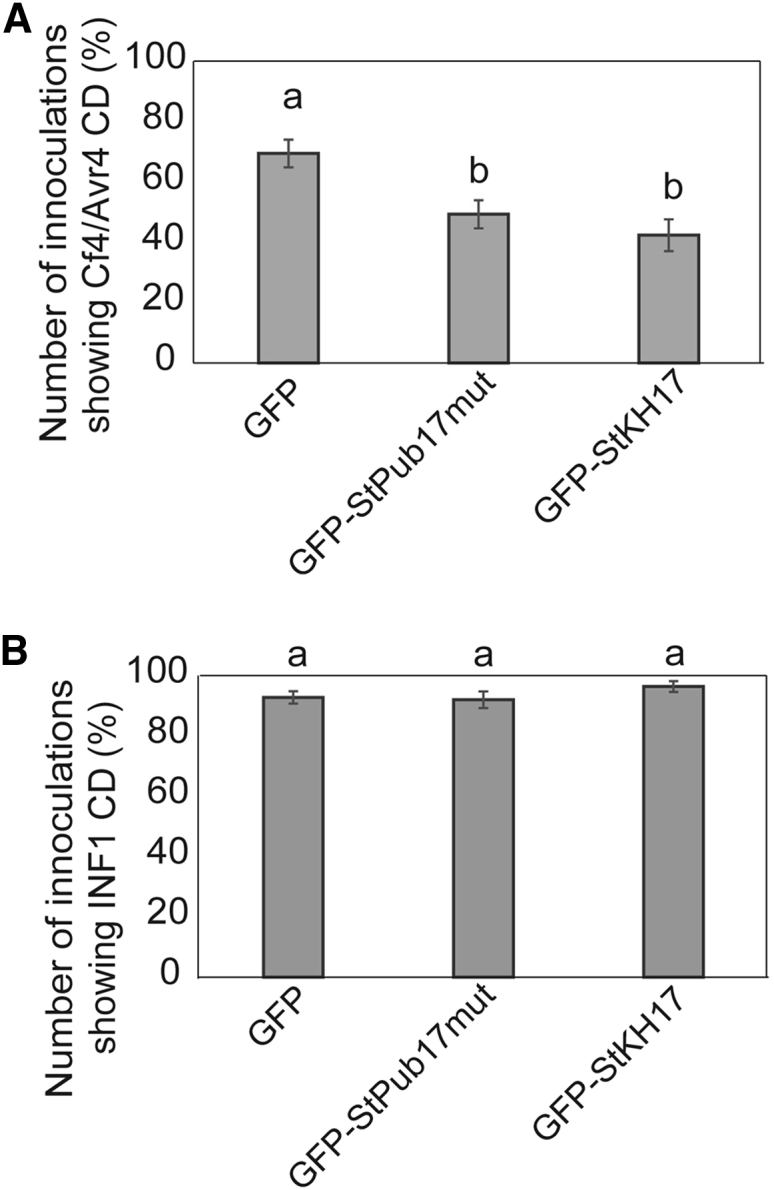
StKH17 Overexpression Suppresses Cf4/Avr4 CD but Not INF1-Triggered CD. **(A)** Overexpression of either GFP-StKH17 or the dominant-negative GFP-StPUB17mut constructs is able to significantly reduce cell death triggered by the recognition of *C. fulvum* Avr4 by tomato Cf4 compared to the overexpression of free GFP (ANOVA p ≤ 0.009, N = 24). The combined data are shown for three biological replicates comprising eight *N. benthamiana* plants per replicate. **(B)** Overexpression of either GFP-StKH17 or the dominant-negative GFP-StPUB17mut constructs has no effect on cell death triggered by the *P. infestans* PAMP INF1 compared to the overexpression of free GFP (ANOVA p = 0.34, N = 24). The combined data are shown for three biological replicates comprising eight *N. benthamiana* plants per replicate. Error bars are SE; lowercase letters indicate significant differences tested by one-way ANOVA with pairwise comparisons performed using the Holm-Sidak test.

A key function StPUB17 plays in immunity is the ability to promote certain cell death responses, such as that triggered by perception of the *Cladosporium fulvum* effector Avr4, by the tomato receptor Cf4 (Yang et al., 2006, He et al., 2015). To explore whether StKH17 is also involved in regulating this immune response, GFP-StKH17 was transiently co-expressed with Cf4 and Avr4 in *N. benthamiana*. The dominant-negative GFP-StPUB17mut, which is able to suppress this cell death (He et al., 2015), was used as a positive control and free GFP was used as a negative control. Both GFP-StKH17 and GFP-StPUB17mut were able to significantly suppress Cf4/Avr4-induced cell death to a similar extent (Figure 5A). As StPUB17 is known to have no involvement in cell death triggered by the *P. infestans* PAMP INF1, GFP-StKH17 was also tested to determine if it regulated this pathway. No significant effect on INF1-triggered cell death was observed following co-expression with either GFP-KH17 or GFP-StPUB17mut (Figure 5B), suggesting that, similar to StPUB17, StKH17 is not involved in this pathway. The fact that both proteins are involved in regulating the same Cf4-associated pathway further supports the hypothesis that StKH17 may be a substrate of StPUB17.

### An Intact RNA Binding Domain Is Required for KH17 to Negatively Regulate Defense

**Figure 6:**
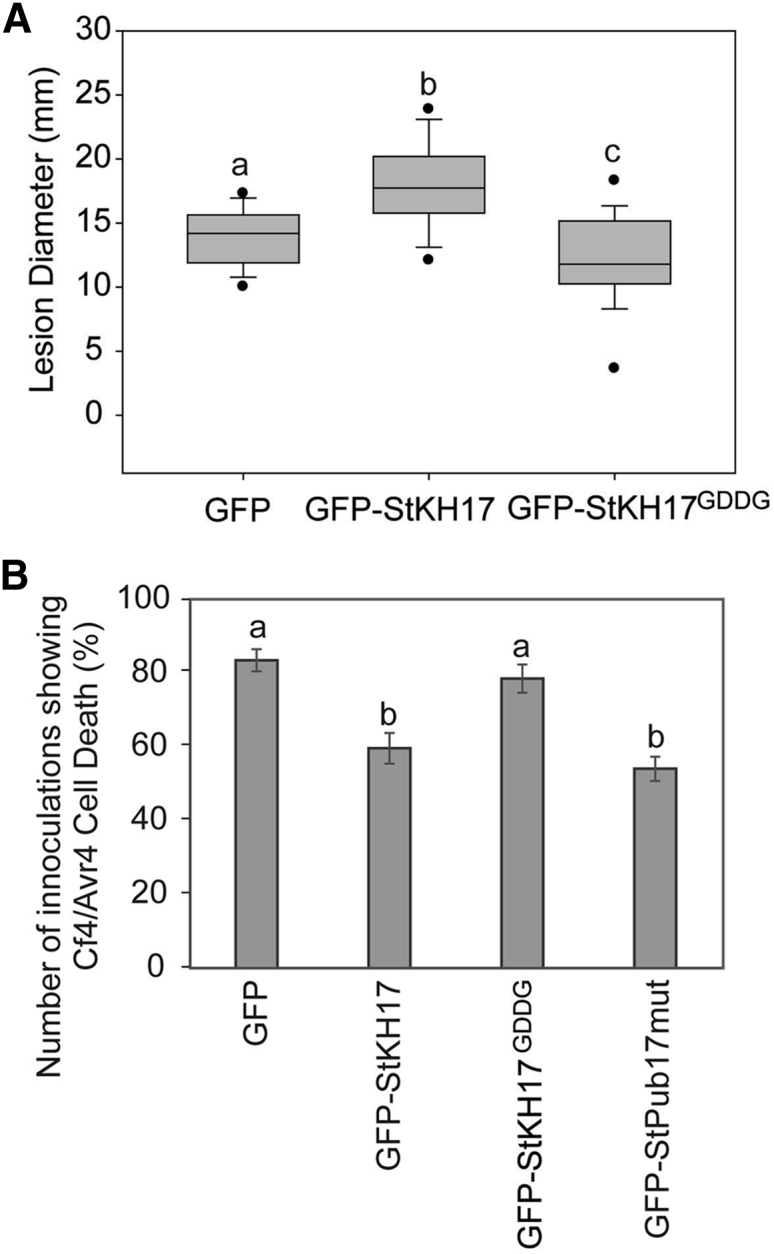
StKH17 Phenotypes Are Dependent on an Intact RNA-Binding Motif. **(A)** Box plot showing the lesion diameter is increased in the halves of leaves transiently overexpressing GFP-StKH17 compared to those overexpressing free GFP, whereas when the RNA-binding motif is mutated to GDDG there is no increase in colonisation observed for GFP-StKH17^GDDG^ (ANOVA p < 0.001, N = 32). The combined data are shown for three biological replicates comprising ~three leaves from ~4 *N. benthamiana* plants per replicate. **(B)** Overexpression of either GFP-StKH17 or dominant-negative GFP-StPUB17mut but not GFP-StKH17^GDDG^ mutant is able to significantly reduce cell death triggered by the recognition of *C. fulvum* Avr4 by Cf4 compared to the overexpression of free GFP (Kruskal-Wallis one-way ANOVA on ranks p ≤ 0.011, N = 45). The combined data are shown for three biological replicates comprising >10 *N. benthamiana* plants per replicate.

KH RBPs typically function by binding RNA through the conserved GxxG motif in the binding cleft and this motif can be mutated to GDDG to prevent RNA binding but maintain protein stability (Hollingworth et al., 2012). In order to investigate the requirement for RNA binding to StKH17 function, the GxxG motif was mutated to GDDG using site-directed mutagenesis (SDM) to give StKH17^GDDG^. Firstly, Y2H analysis was used to show that StKH17^GDDG^ maintains its ability to interact with StPUB17 and StPUB17mut (Supplemental Figure 9A), showing that RNA binding is not required for this interaction. The GFP-StKH17^GDDG^ mutant was also shown to be stable and expressed to similar levels as the WT StKH17 (Supplemental Figure 9B). The GFP-StKH17^GDDG^ mutant also retains the same nuclear localization as WT GFP-StKH17 (Supplemental Figure 9C). However, upon transient overexpression and *P. infestans* infection, GFP-StKH17^GDDG^ is unable to enhance pathogen colonization in the same way as the WT GFP-StKH17 (Figure 6A). In addition, GFP-StKH17^GDDG^ is also unable to suppress Cf4/Avr4-triggered cell death (Figure 6B). Taken together, this suggests that RNA-binding capability is critical for StKH17 to function as a negative regulator of immunity.

## Discussion

Many ubiquitin E3 ligases have been demonstrated to either positively or negatively regulate immunity in plants. Of those that are negative regulators, several substrates have been identified. Some appear to be involved with the regulation of vesicle trafficking through the targeting of GTPase RabA4B, PI4Kβ1/2, and exocyst subunit Exo70B2 for degradation (Stegmann et al., 2012, Antignani et al., 2015). E3 ligases are also known to control the activity of immune-regulating GTPases through targeting the GTPase-activating proteins (GAPs) SPIN6 and RabA4B and guanine nucleotide exchange factor (GEF) SWAP70 for degradation (Antignani et al., 2015, Liu et al., 2015, He et al., 2018). To date, no examples of plant defense-associated RBPs have been identified to be the direct targets of E3 ligases. However, in mammalian systems, the RBP and translational repressor MEX3C contains both a RING-type E3 ligase domain in addition to a KH domain. MEX3C regulates immune responses to viral infection through ubiquitination of receptor RIG1 and the degradation of viral RNA (Kuniyoshi et al., 2014, Yang et al., 2017). The plant E3 ligase SPL11 has been shown to regulate flowering time through ubiquitination of substrate KH RBP SPL11-interacting Protein 1 (SPIN1) indicating that the E3 ligase/RBP combination is a conserved regulatory module (Vega-Sánchez et al., 2008).

**Figure 7:**
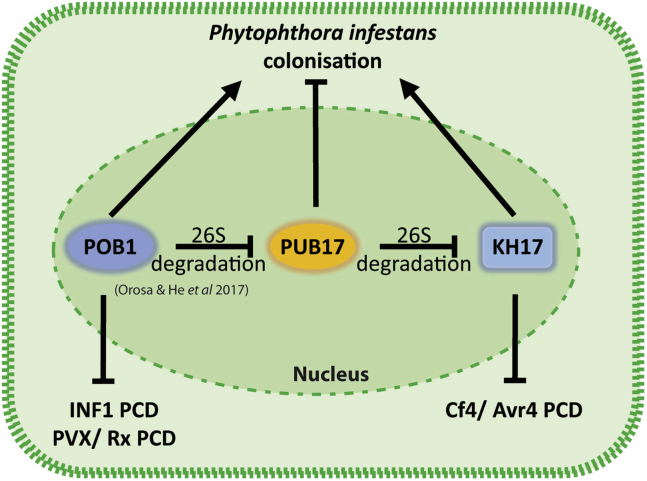
Model Showing the Role of StKH17 in Defense Regulation. The E3 ligase POB1 is a negative regulator of plant defense and overexpression promotes *P. infestans* colonization while negatively regulating INF1 and PVX/Rx PCD. POB1 also negatively regulates Cf4/Avr4 PCD by targeting the E3 ligase StPUB17 for 26S degradation (Orosa et al., 2017). StPUB17 is a positive regulator of immunity and restricts *P. infestans* colonization while promoting Cf4/Avr4 PCD (He et al., 2015). Here the RNA-binding protein StKH17 is shown to be a negative regulator of plant defense and StKH17 overexpression promotes *P. infestans* colonization and negatively regulates Cf4/Avr4 PCD. StPUB17 positively regulates immunity by targeting the negative regulator StKH17 for 26S proteasome degradation.

E3 ligases that negatively regulate immunity have been shown to target positive immune regulators for degradation by the 26S proteasome; for example, POB1 targets StPUB17 for 26S degradation (Orosa et al., 2017). Despite no substrates being identified, the hypothesis is that E3 ligases that positively regulate immunity would target negative regulators. Indeed, StKH17 is shown to be a negative regulator of defense to *P. infestans* as its overexpression expedites pathogen colonization and suppresses Cf4/Avr4-triggered cell death. The model (Figure 7) shows how an E3 ligase cascade regulates specific immune pathways. The E3 ligase POB1 was shown previously to suppress a range of immune responses, including Cf4-mediated cell death. It suppresses Cf4-mediated cell death by targeting the positive regulator PUB17 for degradation (Orosa et al., 2017; Figure 7). In turn, we show in this work that PUB17 targets StKH17 for degradation (Figure 7). Although *Phytophthora* does not trigger Cf4 cell death directly, several effectors from *P. infestans* (Avr3a, PexRD2, Pi22926) have been shown to suppress this pathway, suggesting that it is also triggered by an as yet unidentified *Phytophthora* MAMP (Gilroy et al., 2011, King et al., 2014, Ren et al., 2019). Another example of an RBP that negatively regulates defense is StKBRP1 (Wang et al., 2015). StKRBP1 behaves as a susceptibility (S) factor that is co-opted and stabilized by the activity of the *P. infestans* effector Pi04089 in order to suppress immunity and promote conditions favorable for pathogen colonization. The fact that pathogen effectors have evolved the ability to interact with and manipulate RBPs such as StKRBP1, AtGRP7, and PINP1 (Nicaise et al., 2013, Qiao et al., 2015, Wang et al., 2015) suggests that these proteins are key nodes in the immune signaling regulatory network.

It is unknown why StPUB17 and StPUB17mut accumulate in the nucleolus. The nucleolus is mostly associated with ribosomal RNA synthesis and ribosome biogenesis, although there is new evidence for roles in growth and development, cell cycle, and stress responses (Kalinina et al., 2018). However, it is worth noting that approximately 25% of *P. infestans* RxLR effectors have been shown to have nucleolar localization in the plant cell (Wang et al., 2019), perhaps suggesting that nucleolar processes are targeted by this pathogen. Nuclear localization is clearly required for the immune activity of both POB1 and StPUB17 as fusion to nuclear export signals (NESs) abolished defense-related phenotypes (He et al., 2015, Orosa et al., 2017), but a need for StPUB17 nucleolar localization is unclear. As both the StPUB17 substrate (StKH17) and regulator (POB1) specifically accumulate in the nucleoplasm, this suggests that StPUB17 action occurs in the nucleoplasm rather than the nucleolus (Figure 2; Orosa et al., 2017).

StKH17 stability is clearly reduced in a proteasome-dependent manner in the presence of StPUB17 (Figure 3; Supplemental Figures 4 and 5). As PUB17 is also reduced in stability in a similar manner, it can be argued that the whole complex appears to be turned over by the proteasome. Indeed, there is evidence that E3 ligase stability can be regulated by ubiquitination in either a substrate-dependent or substrate-independent manner. E3 ligases can self-ubiquitinate or be ubiquitinated in *trans* by another ligase (De Bie and Ciechanover, 2011). Autoubiquitination of PUB17 has been demonstrated previously in *in vitro* ubiquitination assays (Yang et al., 2006, He et al., 2015). There was a much smaller reduction in protein stability observed when both StKH17 and StPUB17mut were co-expressed. This is interesting as it has been shown previously in *in vitro* ubiquitination assays that the mutation of PUB17 used in our assays results in a complete loss of E3 ligase activity (Yang et al., 2006). However, PUB E3 ligases are known to be activated by heterodimerizing or homodimerizing via their Ubox domains; auto-ubiquitination is thought to account for the general instability of E3 ligases *in planta* (Trujillo, 2018). The presence of an endogenous WT *Nb*PUB17, either alone or dimerized with the mutant *St*PUB17mut form, could account for the partial reduction in KH17 when co-expressed with the mutant. It is also possible that the PUB17mut-KH17 complex may be partially targeted for degradation by another E3 ligase, such as POB1, which has previously been shown to regulate StPUB17 levels (Orosa et al., 2017). The fact that the StPUB17 mutant retains the same localization and substrate-binding affinity of the WT StPUB17 allows it to act as an efficient dominant-negative protein.

There are a variety of different protein domains in RBPs that are able to recognize and bind in a sequence-specific manner to RNA; these include the KH domain, RRM, or DEAD box helicase domain (Hentze et al., 2018). The KH domain is thought to recognize up to four nucleotides, with binding occurring through the GxxG motif. Many KH RBPs contain multiple KH domains to improve RNA recognition specificity (Hollingworth et al., 2012). However, STAR-type KH RBPs are able to form dimers to enhance recognition specificity (Feracci et al., 2016). Thus, it is possible that StKH17, which contains a STAR domain, may act as a dimer. Moreover, there is evidence that the binding of RNA itself is crucial for the activity of RBPs. AtGRP7 binds to defense-associated *FLS2* and *EFR* transcripts in order to control their stability. ADP-ribosylation of the RRM by HopU1 abolishes this binding, resulting in heightened susceptibility to pathogens (Fu et al., 2007, Nicaise et al., 2013). In the same way, when the GxxG KH RNA-binding motif in StKH17 was mutated to GDDG to disrupt RNA binding, the resulting StKH17^GDDG^ protein was no longer able to promote *P. infestans* colonization or suppress Cf4-triggered cell death.

RBPs are involved in regulating plant defense through the post-transcriptional control of RNA processing, stability, and localization, reviewed in Staiger et al. (2013). This can take many different forms, from control of alternative splicing and nonsense-mediated decay to changes in stability or localization of mRNAs. RBPs such as the argonautes are also involved with small RNA and microRNA generation, targeting, and epigenetic regulation (Staiger et al., 2013). The next steps would be to identify the specific RNAs that are substrates for StKH17 in order to determine its mode of action in the negative regulation of specific immune pathways.

## Materials and Methods

### Plant Materials and Growth

*N. benthamiana* was grown at 22°C in 16-h days with nights at 18°C. Light levels were maintained between 200 and 450 W/m^2^. Potato plantlets were grown *in vitro* in Murashige and Skoog (MS) medium (4% sucrose and 0.7% agar), 3-week-old plantlets were transferred to individual pots in a greenhouse at 20°–26°C with humidity above 80%.

### Cloning

StKH17 was amplified from potato cDNA and attB recombination sites were added using nested PCR; primer sequences are shown in Supplemental Table 1. Gateway entry clones were generated by recombining attB-effector PCR products with pDonr201 and clones were recombined into pB7WGF2 and transferred into *Agrobacterium* for transient assays. The StKH17^GDDG^ mutant was generated using site-directed mutagenesis QuickChange Kit (Stratagene) using pDonr201-StKH17 as a template; primer sequences are shown in Supplemental Table 1.

### Y2H

A screen with StPUB17 was carried out using the Invitrogen ProQuest system and yeast strain MaV203. Briefly, DNA-BD “bait” fusions to StPUB17 were generated using Gateway recombination with an entry clone. This was transformed MaV203 cells and recovered using nutritional selection and tested for reporter gene auto-activation. Competent cells were generated for BD-PUB17 and were transformed with a potato DNA AD “prey” Y2H library. Interacting clones were selected based on the reporter gene activity (i.e., ability to grow on media lacking histidine or uracil and gain of β-galactosidase activity). Interacting clones were sequenced. WT and mutant bait and prey clones were then co-transformed into yeast to test pairwise interactions.

### *P. infestans* Growth

*P. infestans* strain 88069 was grown for 2 weeks at 19°C on Rye agar plates before sporangia were harvested by flooding with sterile distilled water (SDW), scraping with a plastic spreader, and filtering through a 70 μm nylon cell strainer (Corning) to remove hyphae. The resulting suspension was centrifuged at 2750 rpm for 10 minutes and the pellet re-suspended in SDW to 50 000 sporangia per milliliter using a counting chamber.

### *Agrobacterium*-Mediated Transient Infection Assays

*Agrobacterium* strains GV3101 or AGL1 with StKH17 and StPUB17 WT and mutant constructs were grown in yeast extract and beef (YEB) media supplemented with the appropriate antibiotics at 28°C overnight. Cultures were centrifuged at 4000 rpm before resuspension in 10 mM 2-(*N-morpholino)ethanesulfonic acid*: 10 mM MgCl_2_ with 200 μM acetosyringone and adjusted to an optical density 600 (OD_600_) of 0.05 for confocal analysis and 0.5 for western and cell death assays. An OD_600_ of 0.1 was used for *Phytophthora* virulence assays where test and control suspensions were infiltrated in two spots on either half of an *N. benthamiana* leaf (three leaves per plant; six plants per replicate) before being drop inoculated 24 h later with 10 μL of *P. infestans* inoculum at 50 000 sporangia per milliliter and one-way ANOVA was performed to determine statistically significant differences.

### VIGS

VIGS constructs were made by cloning two individual ∼170 bp PCR fragments from NbKH17 into TRV vectors (Ratcliff et al., 2001). *N. benthamiana* is an allotetraploid resulting from the hybridization of two unknown progenitors. It typically contains two similar copies of each gene, one from each parent (Bombarely et al., 2012). Therefore, the VIGS constructs and qPCR primers were designed to knock down and amplify both *NbKH17* genes (*NbKH17a* and *NbKH17b*) respectively. Primer sequences are shown in Supplemental Table 1. A TRV construct expressing GFP was used as a control (He et al., 2015). *Agrobacterium tumefaciens* strains containing a mixture of RNA1 and each NbKH17 VIGS construct at an OD_600_ of 0.5 were infiltrated into the two leaves of the four-leaf-stage *N. benthamiana* plant. Systemic leaves were detached, analyzed by qRT–PCR, and used for *P. infestans* infection 2–3 weeks later. *P. infestans* lesions were measured at 7 days post inoculation (dpi) and sporangia counts were performed at 10 dpi on samples where three leaves were pooled and sporangia recovered in 3 mL of SDW. Counts were carried out using a cell counter and results were analyzed using one-way ANOVA to determine statistically significant differences.

### Quantitative RT–PCR

Total RNA was extracted from the leaves of potato transgenic lines and *N. benthamiana* VIGS plants using a Qiagen RNeasy plant mini kit according to the manufacturer's instructions. The cDNA was synthesized using Invitrogen superscript II kit and qRT–PCR was carried out using SYBR green as described previously (McLellan et al., 2013). Primers for real-time PCR are shown in Supplemental Table 1 and gene expression levels were analyzed using the comparative Ct method as described by Livak and Schmittgen, (2001) and Cikos et al. (2007).

### Generation of Potato Transgenics

*Agrobacterium* containing overexpression vector pK2GW7.0-StKH17 or RNAi vector pHellsgate8-StKH17 were used to transform microtuber discs of the potato cultivar E3 (Tian et al., 2015). Discs were first grown in co-culture medium (3% sucrose MS + 0.2 mg L^−1^ indole-3-acetic acid [IAA]; 0.2 mg L^−1^ gibberellin A3 [GA3]; 0.5 mg L^−1^ 6-benzyl aminopurine [BA]; 2 mg L^−1^ Zeatin [ZT] pH 5.8) before transfer to shoot-generating medium (3% sucrose + MS + 0.2 mg L^−1^ IAA + 0.2 mg L^−1^ GA3 + 0.5 mg L^−1^ 6-BA + 2 mg L^−1^ ZT + 75 mg L^−1^ Kanamycin [Kan]; 200 mg L^−1^ cefalexin [Cef]) and then transferred to root generation medium (3% sucrose, MS + 50 mg L^−1^ Kan; 400 Cef mg l^−1^, pH 5.8). The expression levels of the transgenics was confirmed by qRT–PCR; primers are shown in Supplemental Table 1.

### Confocal Analysis

*A. tumefaciens* containing GFP-StKH17 was pressure infiltrated into leaves of 4-week-old WT *N. benthamiana* plants, separately and together with mRFP-StPUB17mut. Cells expressing fluorescent protein fusions were observed using a Zeiss 710 confocal microscope no more than 2 days post infiltration using a low OD_600_ of 0.05. GFP was excited with a 488 nm laser and the emissions were detected between 500 nm and 530 nm. mRFP was excited with a 561 nm laser and emissions detected between 600 nm and 630 nm. On co-expression, fluorophores were imaged sequentially to minimize cross-talk. Images were processed with propriety confocal software.

### Cell Death Assay

*Agrobacterium* strains (expressing INF1 or Cf4/Avr4) were co-infiltrated into leaves of *N. benthamiana* WT plants with free GFP or GFP-KH17, GFP-KH17^GDDG^, or GFP-StPUB17mut. The number of positive HRs (i.e., more than 50% of the inoculated region produces clear cell death) were counted as described previously (Gilroy et al., 2011) and expressed as the mean percentage of total inoculations per plant. The error bars represent ± SEs of combined data from at least three biological replicates. One-way ANOVA was performed to determine statistically significant differences.

### Western Blotting

Protein fusions were transiently overexpressed for 2dpi in *N. benthamiana* and were tested by western blotting to assess protein presence and stability. Proteins were extracted using GTEN buffer (10% gylcerol; 25 mM Tris pH 7.5; 1 mM EDTA; 150 mM NaCl; 1 mM PMSF; 10 mM DTT; 0.5% Nonidet p40; PI inhibitor tablet) then mixed with 2× SDS–PAGE sample buffer and loaded onto 12% SDS–PAGE gels. Gels were blotted onto nitrocellulose membrane and Ponceau stained to show loading. Membranes were blocked in 4% milk in 1× PBST (137 mM NaCl; 12 mM phosphate; 2.7 mM KCl; pH 7.4; 0.2% Tween-20) before addition of the primary antibodies: a monoclonal GFP antibody at 1:2000 dilution (sc9996; Santa Cruz), a monoclonal anti-cMYC antibody raised in mouse at 1:500 (SC-40; Santa Cruz), a monoclonal anti-RFP antibody produced in rat at 1:4000 (5F8; Chromotek), or a polyclonal ubiquitin antibody produced in rabbit (UBQ11; Agrisera). The membrane was washed with 1× PBST (0.2% Tween 20) five times before addition of the secondary antibody at 1:5000 dilution with anti-mouse Ig-HRP antibody (A9044; Sigma-Aldrich), anti-rat Ig-HRP (ab6836; Abcam), or anti-rabbit Ig-HRP antibody (A8275; Sigma-Aldrich), followed by more washing and ECL (Amersham) development according to the manufacturer's instructions. Relative band intensity was quantified using the Gel Analysis method in ImageJ software.

### Phylogenetic Analysis

Protein sequences were obtained for the following genes: StKH17 (XM_006362273.2; PGSC0003DMT400071249), StKH17-like (XM_006359919.2), NbKH17a (Niben101Scf08926g07008.1), NbKH17b (Niben101Scf09906g02028.1), NbKH17-like a (Niben101Scf02665g15001.1), NbKH17-like b (Niben101Scf00244g03017.1), At2g38610, AT3G08620, and out-grouper StKRBP1 (PGSC0003DMT400066837). CLUSTALW was used to construct an alignment for the full aa sequence. This alignment was imported into TOPOLi v2.5 and a bayesian phylogenetic tree (MrBayes) was constructed.

## Funding

We are grateful for financial support from the 10.13039/501100000268Biotechnology and Biological Sciences Research Council (BBSRC) grants BB/P020569/1, BB/N009967/1, and BB/L026880/1, and the Scottish Government 10.13039/100011310Rural and Environment Science and Analytical Services Division (RESAS). Z.T., K.C., and X.W. were supported by funding from The 10.13039/501100001809National Natural Science Foundation of China (grants 31761143007, 31471550).

## Author Contributions

H.M., P.R.J.B., and Z.T. conceived and designed the experiments. H.M., K.C., Q.H., X.W., and P.C.B. performed experiments and analyzed data. H.M. and P.R.J.B. wrote the manuscript with input from all authors. P.R.J.B. and Z.T. independently secured funding for the research.

## Supplemental Information

Document S1. Supplemental Figures 1–9 and Supplemental Table 1

Document S2. Article plus Supplemental Information
