# Supplementary material for: The Ubiquitin E3 Ligase PUB17 Positively Regulates Immunity by Targeting a Negative Regulator, KH17, for Degradation
Source: Plant Commun. 2020 Jan 7;1(4):100020. doi: 10.1016/j.xplc.2020.100020 (PMC7371183; doi:10.1016/j.xplc.2020.100020)
Supplement: Document S1. Supplemental Figures 1–9 and Supplemental Table 1 [file mmc1.pdf]

Plant Communications, Volume *1*

Supplemental Information

The Ubiquitin E3 Ligase PUB17 Positively Regulates Immunity by Targeting a Negative Regulator, KH17, for Degradation

Hazel McLellan, Kai Chen, Qin He, Xintong Wu, Petra C. Boevink, Zhendong Tian, and Paul R.J. Birch

Supplemental Information

The ubiquitin E3 ligase PUB17 positively regulates immunity by targeting a negative regulator, KH17, for degradation.

Hazel McLellan[1], Kai Chen[2], Qin He[1, 2], Xintong Wu[2], Petra C Boevink[3], Zhendong Tian[2*], Paul RJ Birch[1, 3*]

[1]Division of Plant Science, School of Life Science, University of Dundee (at JHI), Invergowrie, Dundee DD2 5DA, UK.

[2]Key Laboratory of Horticultural Plant Biology (HZAU), Ministry of Education, Key Laboratory of Potato Biology and Biotechnology (HZAU), Ministry of Agriculture and Rural Affairs, Huazhong Agricultural University, Wuhan, Hubei, 430070, China.

[3]Cell and Molecular Science, James Hutton Institute, Invergowrie, Dundee DD2 5DA, UK.

*Authors for Correspondence: Paul.Birch@hutton.ac.uk; tianzhd@mail.hzau.edu.cn

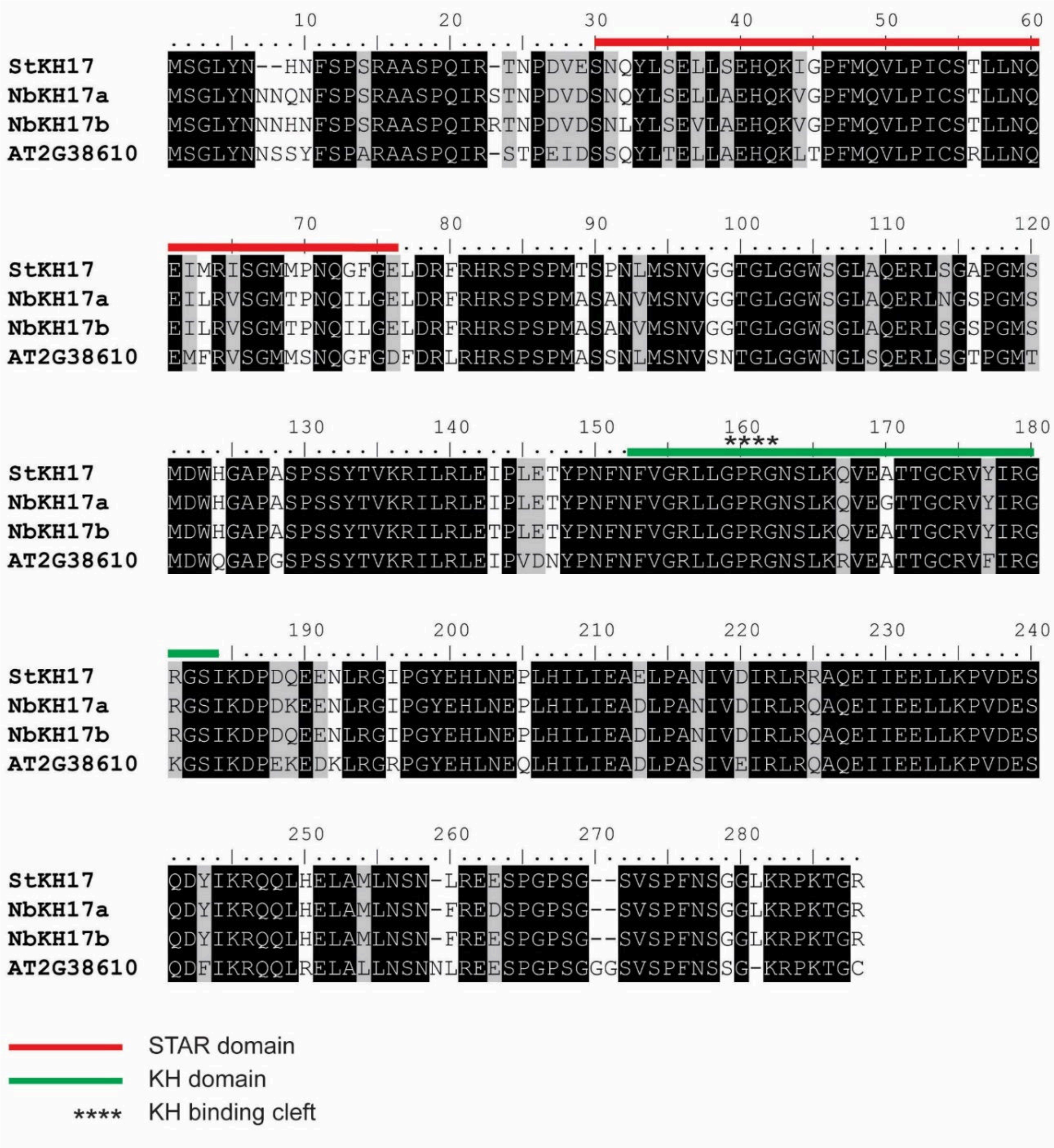

Supplemental Figure S1: Protein alignment of StKH17.

Protein alignment of KH17 orthologues from Potato, *Nicotiana benthamiana* and *Arabidopsis thaliana* constructed using ClustalW. The red line shows the N terminal STAR (signal transducer and activator of RNA) domain which is associated with homodimerisation. The green line shows the KH (K Homology) domain which is associated with RNA binding with the GxxG RNA binding cleft indicated with asterisks.

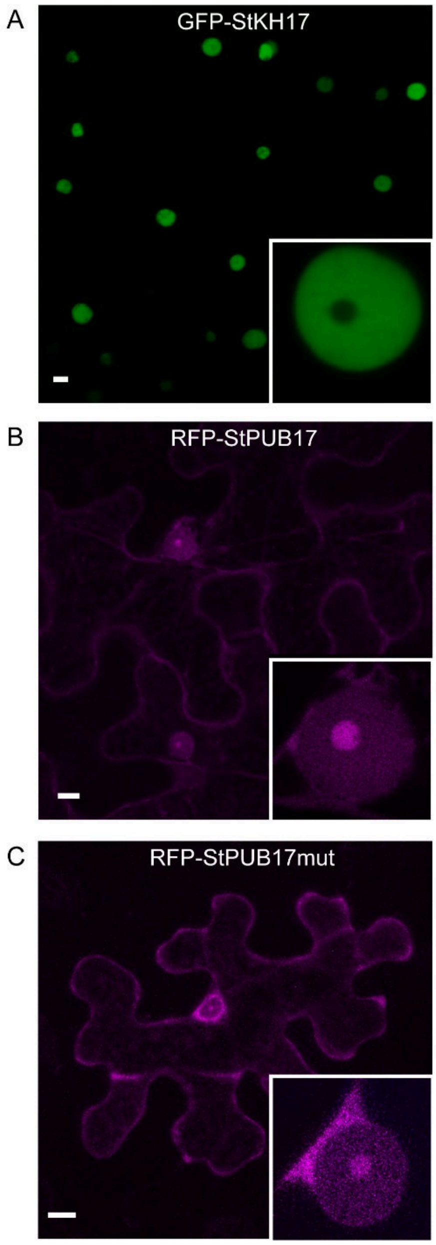

Supplemental Figure S2: Localisation of GFP-KH17 and RFP-Pub17 and RFP-Pub17mut.

Confocal microscopy stacked images showing the subcellular localisations of (A) GFP-KH17, (B) RFP-PUB17 and (C) RFP-PUB17mut as indicated. Magnified images of nuclear slices are shown inset for each construct. GFP (green) and RFP (magenta) channels are shown for the appropriate images. Scale bar is 10 µM.

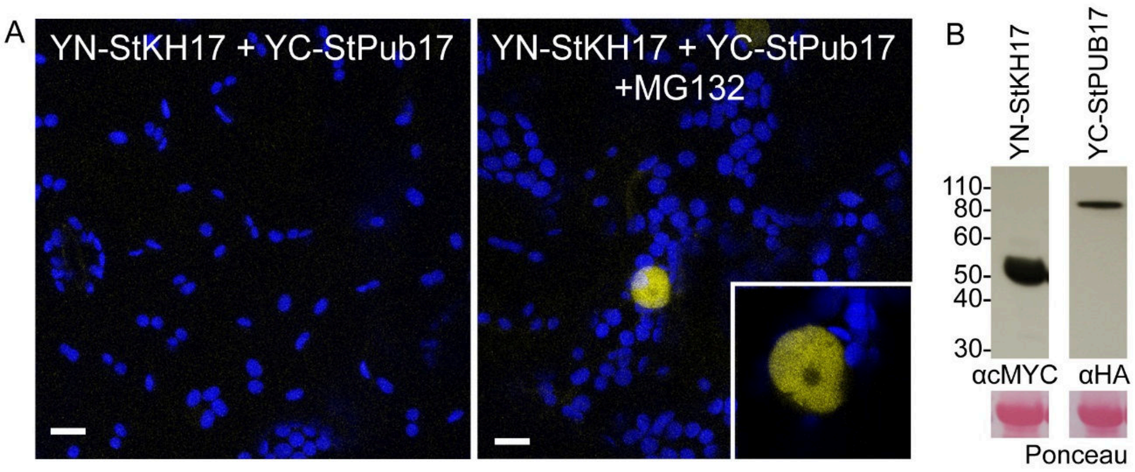

Supplemental Figure S3: Bimolecular Fluorescence complementation of StKH17 and StPUB17

(A) Confocal microscopy stacked images showing BiFC of GFP-StKH17 and RFP-StPUB17 only occurs in the nucleus and only in the presence of proteasome inhibitor MG132. A magnified image of a nuclear slice is shown inset. YFP (Yellow) and Chloroplast auto-fluorescence (blue) channels are merged for each image. Scale bar is 10 μM.

(B) Immunoblot lanes are shown to indicate the stability of the YN-StKH17 and YN-StPUB17 constructs with the appropriate antibodies. Protein size markers are indicated in kilodaltons, and protein loading is indicated by Ponceau stain.

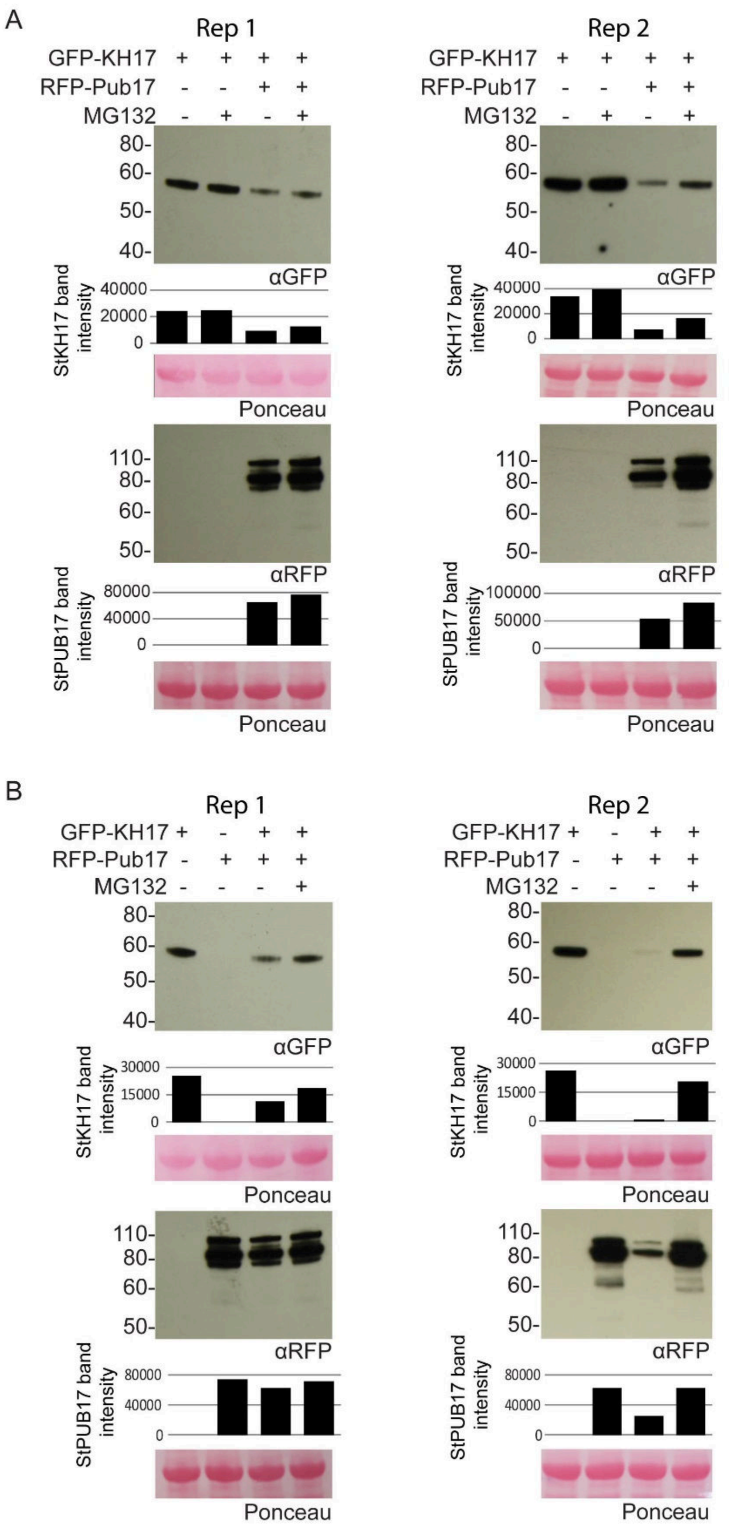

Supplemental Figure S4: StKH17 is turned over in the presence of StPUB17 in a MG132-dependent manner.

(A) Independent biological replicate (Rep) immunoblots showing that the stability of both GFP-StKH17 and RFP-StPUB17 is reduced upon their co-expression and that protein stability is at least partially recovered following MG132 treatment. Graphs show band intensity measurements corresponding to the immunoblot panel directly above.

(B) Independent biological replicates (Rep) showing that the stability of both GFP-StKH17 and RFP-StPUB17 is reduced upon their co-expression compared to when each is expressed alone. The reduced protein stability is at least partially recovered following MG132 treatment. Graphs show band intensity measurements corresponding to the immunoblot panel directly above.

Expression of constructs or treatment for 6 hours with 100 µM MG132 is indicated by a “+.” Protein size markers are indicated in kilodaltons, and protein loading is indicated by Ponceau stain.

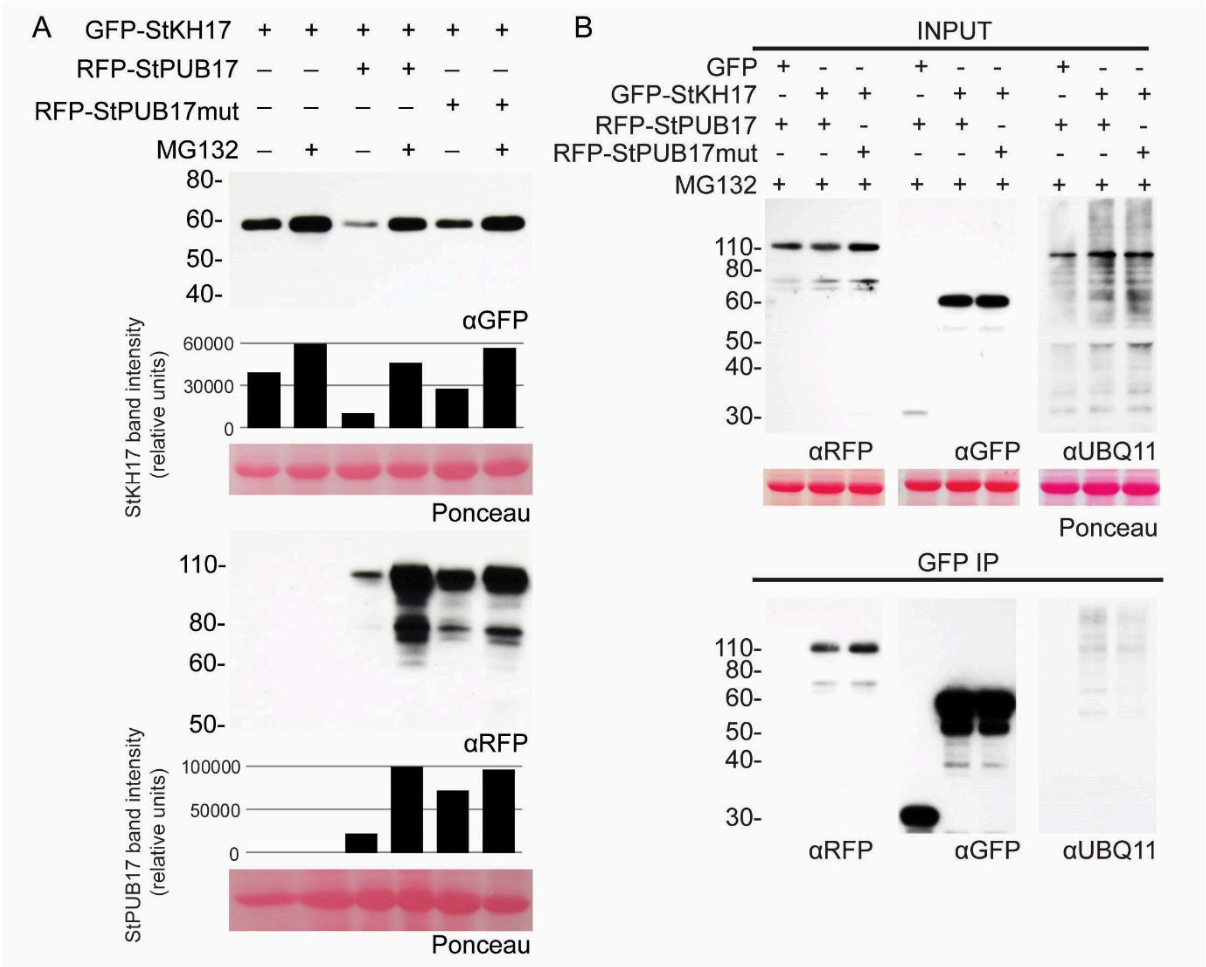

Supplemental Figure S5: StKH17 ubiquitination and turnover by StPUB17 is reduced in the presence of StPUB17mut.

(A) Independent additional replicate to Figure 3A immunoblot showing that the stability of both GFP-StKH17 and RFP-StPUB17 WT (and to a lesser extent RFP-StPUB17mut) is reduced upon their co-expression and that protein stability is recovered following MG132 treatment. Graphs show band intensity measurements corresponding to the immunoblot panel directly above.

(B) Independent additional replicate ubiquitination assay immunoblots showing strong ubiquitin laddering of GFP-StKH17 in the presence of RFP-StPUB17 following IP with GFP trap beads, this ubiquitination is much weaker when GFP-StKH17 is co-expressed with RFP-StPUB17mut. Free GFP is not ubiquitinated by RFP-StPUB17.

Expression of constructs or treatment for 6 hours with 100 µM MG132 is indicated by a "+." Protein size markers are indicated in kilodaltons, and protein loading is indicated by Ponceau stain.

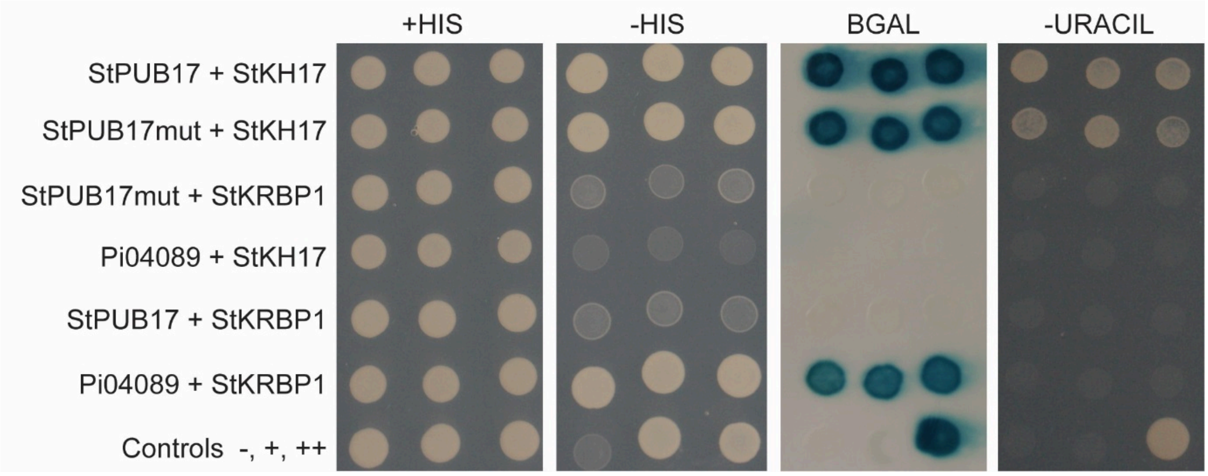

Supplemental Figure S6: Interaction of StPUB17mut with StKH17.

Yeast containing StPUB17 or StPUB17mut and StKH17 grew on medium lacking histidine (-HIS) or uracil and showed β-galactosidase (BGAL) activity indicating protein-protein interaction. Yeast co-expressing controls Pi04089 and StKRBP1 grow on –HIS and show BGAL activity but there was no activation of any reporters when either was co-expressed with StKH17 or StPUB17mut. All yeast grew on medium containing histidine (+HIS). The yeast controls are as follows - = no interaction, + = weak interaction, ++= strong interaction.

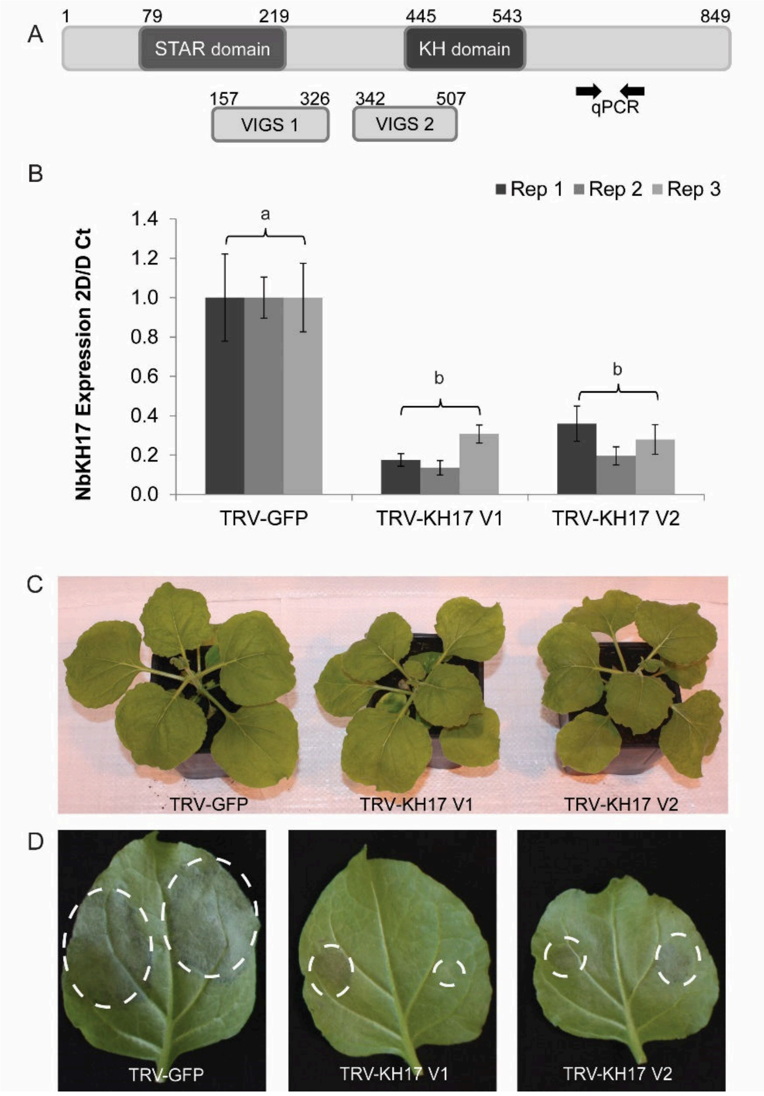

Supplemental Figure S7: VIGS of KH17.

(A) Schematic diagram of NbKH17 showing the positions of the STAR and KH domains alongside the areas used for VIGS constructs and qPCR. Numbers indicate nucleotide residues.

(B) Graph shows 3 independent biological replicates of qRT-PCR data showing the reduction in expression of KH17 expression in the two KH17 VIGS constructs, compared to the TRV-GFP control (ANOVA $P<0.001$). Data was analysed using the 2 Delta-delta Ct method (Livak & Schmittgen, 2001) with *NbEF1a* used as a control. Expression levels in TRV-GFP were set to 1 and other samples were made relative to this. Error bars are standard error, lowercase letters indicate significant differences tested by one way ANOVA with pairwise comparisons performed using the Holm-sidak test.

(C) Representative images of the TRV-KH17 VIGS plants compared to the TRV-GFP control showing that there were no obvious developmental phenotypes observed in these plants.

(D) Representative leaf infection images of TRV-KH17 VIGS plants compared to the TRV-GFP control. Lesion borders are shown with a white dashed line.

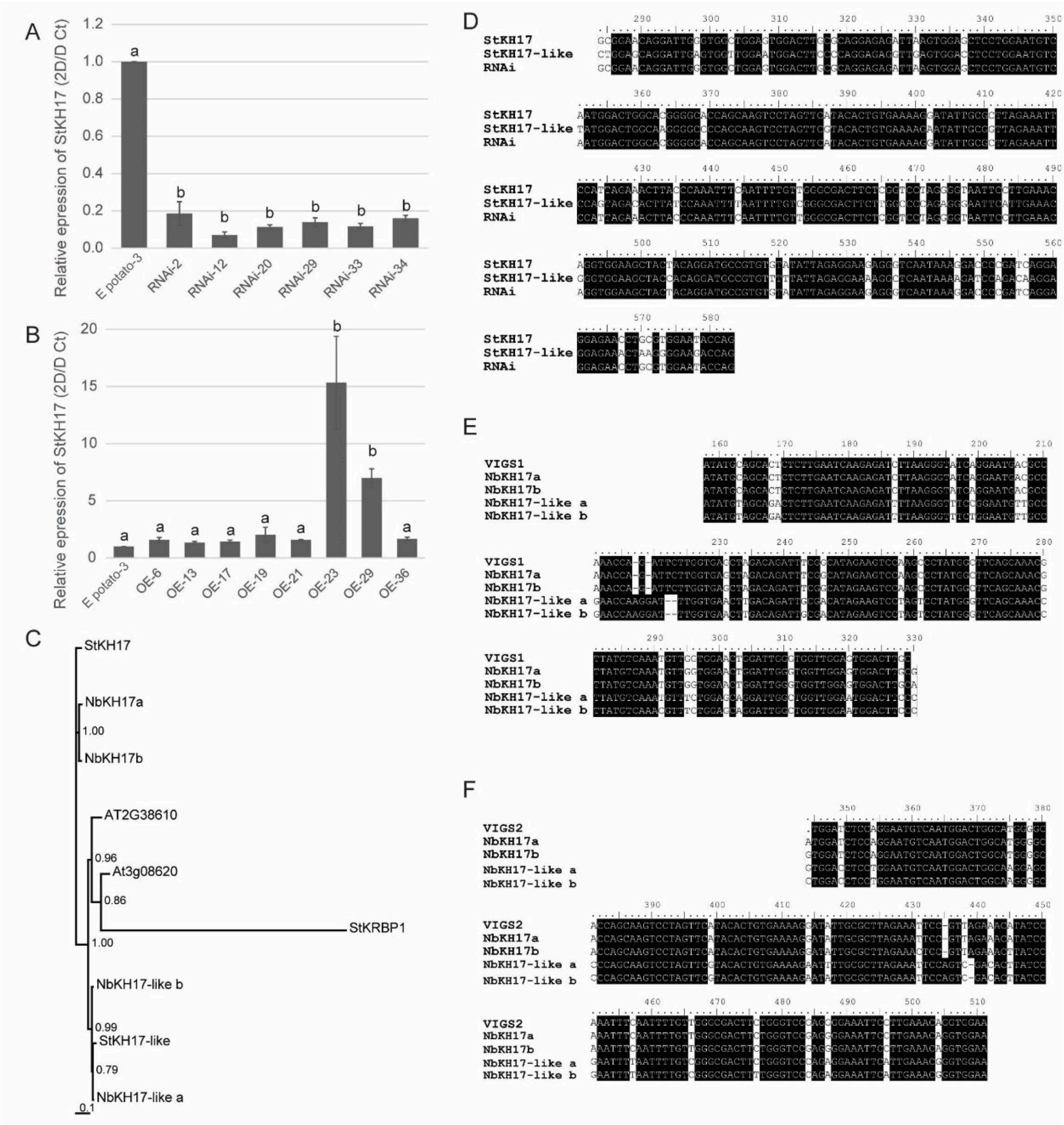

Supplemental Figure S8: Expression levels of *StKH17* in potato transgenic plants.

(A) Graph shows *StKH17* expression levels in independent potato transformants made to stably silence *KH17* using RNAi compared to the expression in the control E3 (ANOVA P<0.001).

(B) Graph shows *StKH17* expression levels in independent potato transformants made to stably overexpress (OE) *StKH17* compared to the expression in the control E3 (Kruskal-Wallis One Way Analysis of Variance on Ranks P = 0.010).

Data was analysed using the 2 Delta-delta Ct method (Livak & Schmittgen, 2001) with *StEf1α* used as a housekeeping gene. Expression levels in E3 plants were set to 1 and other samples were made relative to this. Error bars are standard error; lowercase letters indicate significant differences.

(C) Phylogenetic Bayesian tree showing that the *KH17* and *KH17-like* protein sequences in the solanaceae group separately from each other and the *Arabidopsis* orthologues. Scale bar shows 0.1 substitutions.

(D) Nucleotide alignment of *StKH17, StKH17-like* and the potato StKH17 RNAi construct showing no 21 nt hits to *StKH17-like* and thus no potential off-target silencing.

(E) Nucleotide alignment of *NbKH17a* and *b*, *NbKH17-like a* and *b* and the *N. benthamiana* VIGS1 construct showing no 21 nt hits to *NbKH17-like a* and *b* and thus no potential off-target silencing.

(F) Nucleotide alignment of *NbKH17a* and *b*, *NbKH17-like a* and *b* and the *N. benthamiana* VIGS2 construct showing no 21 nt hits to *NbKH17-like a* and *b* and thus no potential off-target silencing.

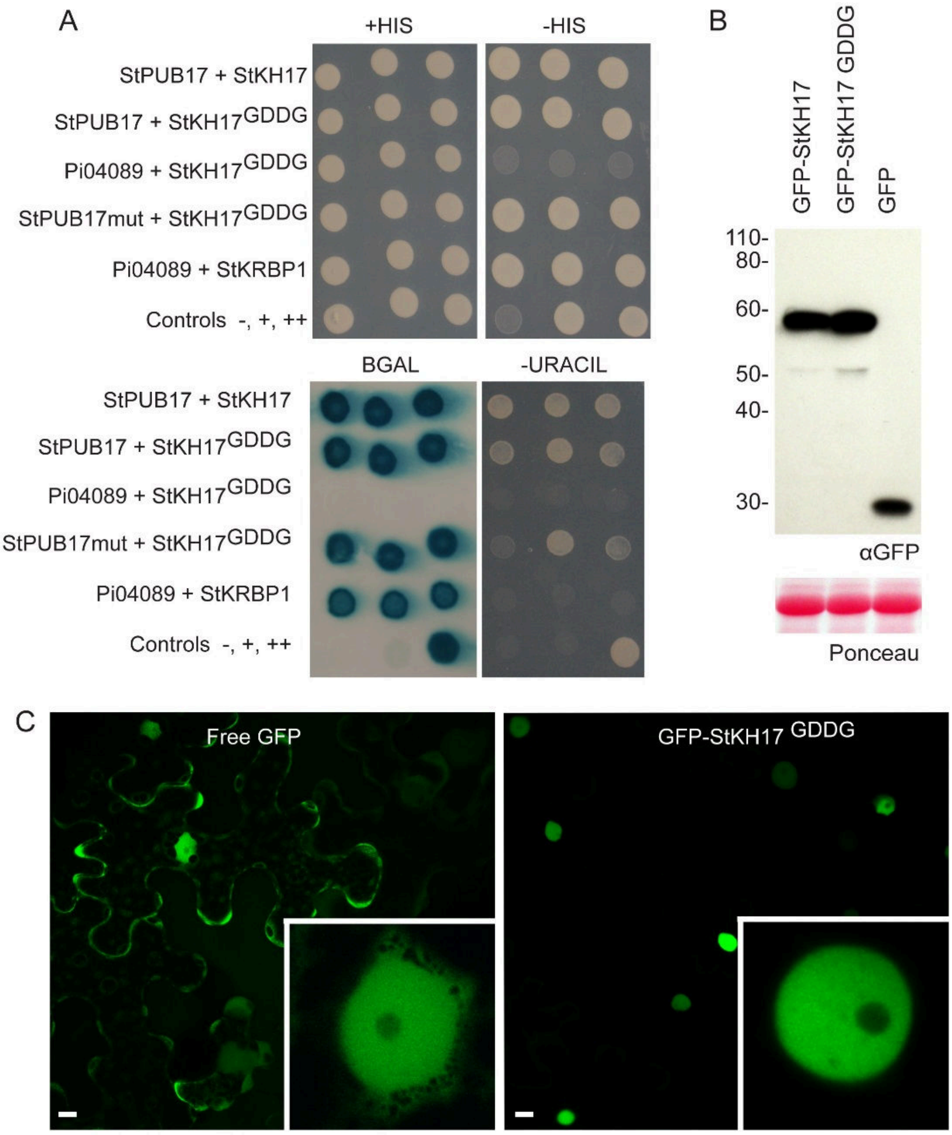

Supplemental Figure S9: StKH17^{GDDG} maintains protein-protein interactions, is stable and localises to the nucleus.

(A) Yeast containing StPUB17 or StPUB17mut and StKH17 or StKH17^{GDDG} mutant grew on medium lacking histidine (-HIS) or uracil and showed β-galactosidase (BGAL) activity indicating protein-protein interaction. Yeast co-expressing controls Pi04089 and StKRBP1 grow on –HIS and show BGAL activity but there was no activation of any reporters when Pi04089 was co-expressed with StKH17^{GDDG} mutant. All yeast grew on medium containing histidine (+HIS). The yeast controls are as follows - = no interaction, + = weak interaction, ++= strong interaction.

(B) Immunoblot showing the comparable stability of GFP-StKH17 and GFP-StKH17^{GDDG}. Protein size markers are indicated in kilodaltons, and protein loading is indicated by Ponceau stain.

(C) Confocal microscopy stacked images showing the subcellular localisations of free GFP and GFP-KH17^{GDDG} mutant as indicated. Magnified images of nuclear slices are shown inset for each construct. The GFP (green) channel is shown for both images. Scale bar is 10 μM.

Supplemental Table S1: Primers used in this work.

Gene	Primer	Use
StKH17	GW_StKH17_F: AAAGCAGGCTTCACCATGTCAGGTTTATATAATC<br>GW_StKH17_R: GAAAGCTGGGTCTCAACGACCAGTCTTGGGAC	Gateway cloning
NbKH17	KH17_V1F: CCCCGAATTCATATGCAGCACTCTCTTGAATC<br>KH17_V1R: CCCCGTTAACGCAAGTCCACTCCAACCAC	VIGS cloning
NbKH17	KH17_V2F: CCCCGAATTCTGGATCTCCAGGAATGTCAA<br>KH17_V2R: CCCCGTTAACTTCCACCTGTTTCAAGGAATTT	VIGS cloning
StKH17	RNAiF: GTACAAAAAAGCAGGCTGCGGAACAGGATTGGGTG<br>RNAiR: CTTTGTACAAGAAAGCTGGGTCTGGTATTCCACGCAGGTTC	Potato RNAi cloning
StKH17	StKH17F:AAAAAAGCAGGCTTCATGTCAGGTTTATATAATCATAACTTTTC<br>StKH17R: CAAGAAAGCTGGGTTCAACGACCAGTCTTGGG	Potato OE cloning
StKH17	StKH17mF:TCGGTgacgacGGTAATTCCTTGAAACAGGTGGA<br>StKH17mR:AATTACCgtcgtcACCGAGAAGTCGCCCAACAA	GDDG mutation
StEF1α	StEF1αF: ATTGGAAACGGATATGCTCCA<br>StEF1αR: TCCTTACCTGAACGCCTGTCA	qRT-PCR
StKH17	qRT-StKH17F: TTTGTTGGGCGACTTCTCGG<br>qRT-StKH17R: CCTGGTATTCCACGCAGGTT	qRT-PCR
NbEf1α	qRT-Nb-ef1aF: TGGACACAGGGACTTCATCA<br>qRT-Nb-ef1aR: CAAGGGTGAAAGCAAGCAAT	qRT-PCR
NbKH17	qRT-NbKH17F: AGGCAGATTTACCAGCGAAC<br>qRT-NbKH17R: CCACTGGCTTGAGCAACTC	qRT-PCR
